# β-Alanine ingestion increases muscle carnosine content and combat specific performance in soldiers

**DOI:** 10.1007/s00726-014-1896-7

**Published:** 2014-12-16

**Authors:** Jay R. Hoffman, Geva Landau, Jeffrey R. Stout, Mattan W. Hoffman, Nurit Shavit, Philip Rosen, Daniel S. Moran, David H. Fukuda, Ilan Shelef, Erez Carmom, Ishay Ostfeld

**Affiliations:** 1 Institute of Exercise Physiology and Wellness, Sport and Exercise Science, University of Central Florida, Orlando, FL 32816 USA; 2 Israel Defense Force, Medical Corps, Air Force Medical Center, Tel Hashomer, Israel; 3 Israel Defense Forces, Combat Fitness Branch, Netanya, Israel; 4 Soroka Medical Center, Beersheva, Israel; 5 Center of Samaria, School of Health Science, Ariel University, Ariel, Israel; 6 Israel Defense Force, Medical Corps, Tel Hashomer, Israel

**Keywords:** Histidine-related compounds, Amino acids, Exercise, Supplements

## Abstract

The purpose of this study was to examine the effect of β-alanine (BA) ingestion on tissue carnosine levels and the impact such changes would have on combat specific activity. Eighteen soldiers (19.9 ± 0.8 year) from an elite combat unit were randomly assigned to either a BA or placebo (PL) group. Before and following a 30-day supplementation period carnosine content of the gastrocnemius muscle and brain was determined by proton magnetic resonance spectroscopy. During each testing session, participants performed military relevant tasks that included a 2.5 km run, a 1-min sprint, 50-m casualty carry, repeated 30-m sprints with target shooting, and a 2-min serial subtraction test (SST) to assess cognitive function under stressful conditions. A significant elevation (*p* = 0.048) in muscle carnosine content was noted in BA compared to PL. Changes in muscle carnosine content was correlated to changes in fatigue rate (*r* = 0.633, *p* = 0.06). No changes (*p* = 0.607) were observed in brain carnosine content. Following supplementation, no differences were noted in 2.5 km run, 1-min sprint, repeated sprint, or marksmanship performance, but participants in BA significantly (*p* = 0.044) improved their time for the 50-m casualty carry and increased their performance (*p* = 0.022) in the SST compared to PL. In summary, 30-days of BA ingestion can increase muscle carnosine content and improve aspects of military specific performance. Although cognitive performance was significantly greater in participants consuming BA compared to placebo, current study methods were unable to detect any change in brain carnosine levels, thus, the precise mechanism underlying these effects remains elusive.

## Introduction

Increases in the concentration of histidine containing dipeptides within skeletal muscle have been demonstrated to have ergogenic potential by increasing the muscles ability to buffer elevations in hydrogen ion (H+) concentrations (Harris et al. 1990). Carnosine, anserine, balenine, and homocarnosine are histidine containing dipeptides that are widely distributed in vertebrate tissue including skeletal muscle, heart and brain. Carnosine, anserine and balenine are comprised of the amino acids l-histidine and β-alanine and differ in regards to where the methylation on the imidazole ring appears on the l-histidine molecule (carnosine is non-methylated, while anserine and balenine are methylated) (Boldyrev et al. 2013). It is the imidazole ring of the histidine containing dipeptide that regulates buffering activity. However, only carnosine is found in human skeletal tissue, with small concentrations also reported in the olfactory bulb of the brain (Boldyrev et al. 2013). Considering that both histidine and carnosine synthetase (the enzyme responsible for the synthesis of carnosine) are found in high concentrations in skeletal muscle, it is believed that β-alanine is the rate-limiting step in carnosine formation (Derave et al. 2010).

β-Alanine can be endogenously synthesized in small concentrations by hepatic uracil degradation. However, it is not synthesized in sufficient concentrations to cause an increase in muscle carnosine synthesis (Harris et al. 2006). Dietary ingestion of β-alanine may result in a transient elevation in muscle carnosine synthesis, but it does not provide the consistent transport of β-alanine into the muscle that will result in significant changes in muscle carnosine concentrations (Harris et al. 2006). Evidence has been quite convincing that when β-alanine is provided as a dietary supplement for 4–10 weeks, significant increases in muscle carnosine concentrations occur (Baguet et al. 2009, 2010; Harris et al. 2006; Hill et al. 2007). Further, elevations in muscle carnosine are associated with significant performance improvements during high-intense physical activity (Hill et al. 2007; Hoffman et al. 2008; Van Thienen et al. 2009).

Scientific examination of β-alanine supplementation has focused on its ability to increase muscle carnosine concentrations and enhance muscle buffering capacity. However, carnosine appears to have a broader biological role, and has been proposed to serve as an antioxidant, antiglycating and ion-chelating agent suggesting that it may have a potential role as a neuro-protector during oxidative stress (Boldyrev et al. 2010; Hipkiss et al. 1998; Kohen et al. 1988). However, only one study is known that has examined the effect of β-alanine ingestion on changes in carnosine concentrations in the brain, and this study examined a rodent model. Murakami and Furuse (2010) reported an increase of carnosine concentrations in the cerebral cortex and hypothalamus in mice provided with a daily ingestion of β-alanine under stressful conditions. Increases in brain carnosine concentrations were also associated with elevations in brain-derived neurotrophic factor (BDNF) and a decrease in 5-hydroxyindoleacetic acid concentrations (a metabolite of serotonin) in the hippocampus. In addition to the changes in brain metabolites, the authors also reported an improved time in a maze containing anxiety producing compounds, suggesting that β-alanine ingestion may have anxiolytic-like effects (Murakami and Furuse 2010). To date, changes in brain carnosine concentrations from β-alanine ingestion in a human study has not been examined.

The lack of investigations focusing on increases in brain carnosine concentrations is likely related to the previous necessity to use invasive techniques to measure carnosine concentrations (e.g., biopsy). However, recent technological advances using magnetic resonance spectroscopy (MRS) has resulted in an effective and valid method to measure carnosine concentrations in tissue (Özdemir et al. 2007). As discussed earlier, carnosine content in the human brain has been found only in the olfactory bulb (Boldyrev et al. 2013), and present understanding suggests that the major histidine dipeptide found in the human brain is homocarnosine (Boldyrev et al. 2013). Homocarnosine is an analog of carnosine, in which the β-alanine molecule is replaced by *γ*-aminobutyric acid (GABA). Limitations with MRS technology though make it difficult to differentiate between carnosine and homocarnosine (Boldyrev et al. 2013). However, if β-alanine is provided as a supplement, while GABA ingestion remains similar for all participants, it can be postulated that any changes noted in the spectrum would be due to an increase in carnosine and not homocarnosine.

If brain carnosine concentrations can be shown to increase in humans, it may provide a benefit in maintaining focus, alertness and cognitive function during highly fatiguing, high intense activity such as that seen in both competitive and tactical athletes. During prolonged, high-intensity military training or simulated combat exercises, significant decreases in physical and cognitive performance measures are often reported (Lieberman et al. 2002; Nindl et al. 2007). In a previous study that has examined the effects of β-alanine supplementation in military personnel, Hoffman et al. (2014) demonstrated that 4-weeks of β-alanine ingestion can enhance jump power performance, marksmanship and target engagement speed. These improvements occurred following 4-weeks of highly intense training and followed an acute fatiguing event. However, those investigators did not measure changes in muscle or brain carnosine concentrations. Thus, the purpose of this study was to examine the effect of 30-days of β-alanine ingestion on both muscle and brain carnosine concentrations and the impact such changes would have on combat specific activity.

## Methods

### Subjects

Eighteen male soldiers from an elite combat unit of the Israel Defense Forces (IDF) volunteered to participate in this double-blind study. Following an explanation of all procedures, risks and benefits, each participant provided his informed consent to participate in the study. The Helsinki committee of the IDF Medical Corp approved this research study. Subjects were not permitted to use any additional dietary supplementation and did not consume any androgens or any other performance enhancing drugs. Screening for performance enhancing drug use and additional supplementation was accomplished via a health questionnaire completed during participant recruitment. Participants were from the same unit and were randomly assigned to one of two groups. The randomization procedure involved alternating group assignment of volunteers. Using the procedures described by Gravetter and Wallnau (1996) for estimating samples sizes for repeated measures designs, a sample size of 9 of each group resulted in a statistical power (1−β) of >0.90 based on the changes in sprint performance reported by Van Thienen et al. (2009). The first group; (BA; *n* = 9; age 19.6 ± 0.5 years; height 1.76 ± 0.05 m; body mass 72.1 ± 4.5 kg) consumed 6.0 g of β-alanine per day, while the second group (PL; *n* = 9; age 20.2 ± 1.0 years; height 1.79 ± 0.08 m; body mass 76.4 ± 6.1 kg) consumed 6.0 g of placebo (rice flour). During the 30-day study period all participants participated in the same advanced military training tasks that included combat skill development, physical work under pressure, navigational training, self-defense/hand-to-hand combat and conditioning.

### Testing protocol

This randomized, double-blind, placebo-controlled investigation was conducted at the unit’s training facilities, under the unit’s regular training protocols and safety regulations. Data collection occurred before (PRE) and following (POST) 30-days of supplementation. During each session participants performed military relevant tasks that included a 2.5 km run, a 1-min sprint, and a 50-m casualty carry. In addition, participants performed repeated 30-m sprints in combat gear (combat vest with ammunition, helmet, and assault rifle). Between each sprint soldiers were tested on marksmanship. Immediately following the final sprint and target shooting, participants performed a 2-min serial subtraction test on the firing range to assess cognitive function under stressful conditions (continuous shooting). These assessments were based upon previously published investigations examining military performance responses during stressful conditions (Harman et al. 2008; Nindl et al. 2002).

### Performance measurements

#### Muscle and brain carnosine content

Carnosine content of all participants was assessed by proton magnetic resonance spectroscopy (MRS) in the gastrocnemius muscle and the brain. All studies were performed on a 3-T system (Ingenia, Philips Medical Systems, Best, The Netherlands). Single-voxel, STEAM acquisitions of the gastrocnemius medialis muscle of the lower leg were carried out using a transmit-receive (16-channel) coil, and in the parieto-occipital lobe of the brain using a 32-channel receive-only coil. In the leg, the scan parameters were TR/TM/TE = 2000/12/13 ms, and in the brain 2000/9.4/16 ms. Second-order shimming was used giving a full-width-half-maximum line width of approximately, 25 Hz in the calf muscle and 12 Hz in the head. Water suppression was achieved by applying two bandwidth selective rf pulses. The average voxel size for the muscle spectra was 35 × 18 × 58 mm (AP × RL × FH) and 39 × 41 × 45 mm (AP × RL × FH) in the brain. The spectral resolution for all spectra was 0.96 Hz, and 400 averages were acquired for a scan time of 13:56 min. The spectra were analyzed using the Philips SpectroView software.

A 1-l solution of 20 mM l-carnosine (Sigma–Aldrich) in 0.1 M potassium phosphate buffer (pH 7.2) was used as an external reference phantom for absolute quantification. The following equation was used (Baguet et al. 2010; Derave et al. 2010) to determine the concentration of carnosine in the gastrocnemius muscle using the C2-H peak at ~8.0 ppm:

$$\left[ {C_{m} } \right] = [C_{r} ] \times \frac{{S_{m} V_{r} C_{T1r} C_{T2r} T_{m} }}{{S_{r} V_{m} C_{T1m} C_{T2m} T_{r} }}$$

where [C_*m*_], [C_*r*_] are the l-carnosine concentrations in vivo and the reference phantom, respectively; S_*m*_, S_*r*_ are the estimated peak areas of the C2-H carnosine peak in vivo and the reference phantom, respectively; V_*m*_, V_*r*_ are the volumes of the voxels in vivo and in the reference phantom, respectively; C_T1m_, C_T1r_, C_T2m_, C_T2r_ are the correction factors for the T1 and T2 relaxation times in vivo and in the reference phantom, respectively; T_*m*_, T_*r*_ are the temperatures (°K) in vivo and in the reference phantom, respectively. The formulae used to calculate the correction factors were those previously recommended (Baguet et al. 2010; Derave et al. 2010):

$$C_{T1} = [1 - \exp ( - TR/T1)]$$

$$C_{T2} = \exp ( - TE/T2)$$

The T1, T2 values for the phantom and muscle were taken from Baguet et al. (2010). The correction for the coil loading was calculated according to the method previously described (Soher et al. 1996).

In the brain, the region of the C2-H resonance of carnosine at 7.9–8.0 ppm also contains contributions from the proton of the n-aspartylaceteate amide group (7.9 ppm) and broad resonances from the exchangeable protons from purine nucleosides and nucleotides at 8.3 ppm. Therefore, changes in the carnosine concentration can only be detected by subtraction of the spectra before and after supplement or placebo ingestion (Vermathen et al. 2000).

#### 2.5 km run and 1-min sprint

These tests simulated a rapid approach to the battlefield. Previous research has suggested that a prolonged run with sprint is a standard approach to the battlefield (Harman et al. 2008). During the run and sprint all participants were dressed in shorts, T-shirt and running shoes. Both the run and sprint were performed on an asphalt road. All participants were provided with an individual global positioning system (GPS) that they wore in a vest underneath their shirt. The GPS unit (MinimaxX, V4.3, Catapult Innovations, Victoria, Australia) was positioned in a posterior pocket on the vest situated between the participant’s right and left scapula in the upper-thoracic spine region. Information on velocity patterns was recorded during the 2.5 km run, as well as total distance run during the 1-min sprint. During the 2.5 km run the velocity of the run was divided into three operationally distinct thresholds and defined as low speed (2.50–3.60 ms^−1^), moderate speed (3.61–4.43 ms^−1^) or high speed (>4.44 ms^−1^). In addition, the average velocity and average heart rate during the 2.5 km run were also downloaded from the GPS receiver/transmitters.

During the 1-min sprint peak velocity, average velocity, total distance, total distance within 90 % of peak velocity, and percent decline was downloaded from the GPS receiver/transmitters. All data were collected at 10 Hz and all analyses were performed with the system software provided by the manufacturer. The validity and reliability of the GPS technology has been previously demonstrated (Varley et al. 2012).

#### 50-m Casualty carry

This test simulated the rescue of a wounded soldier on the battlefield. This test was a modified version of that previously reported (Harman et al. 2008). All participants began the test with a 60 kg manikin on their back, using a “fireman’s carry”. On a verbal command the participant sprinted with the manikin to a cone 25-m away and returned to the starting position. All sprints were performed on a sand and dirt surface. All timing was performed with a stopwatch that measured time to the nearest 1/100th of a second. The same investigator conducted all sprint trials during PRE and POST testing.

#### Repeated sprints and shooting performance

This test mimicked the repeated sprints and shooting engagement often encountered on an urban battlefield. The short sprints mimic the repeated rushes between points of cover during a combat situation (Harman et al. 2008). Each participant began in a two point stance at the edge of the firing range in full combat gear (combat vest with ammunition, helmet, and assault rifle). Upon a verbal command the participant sprinted around a cone 15-m away and returned to the firing range. Each participant sprinted to a designated spot and lay prone on the ground as quickly as possible and delivered three shots to a target 30-m away. All targets were headshots and each shot that hit the target was considered accurate. Participants had 5-s to deliver three shots to the target. Upon completion of the three shots each participant pivoted and returned to the starting line and repeated the sprint and shooting sequence. A total of five sprint and shooting rounds were completed (a total of 15 shots were delivered onto the target). For safety purposes participants did not sprint with their assault rifle. All sprints were performed on a sand and dirt surface. All timing was performed with a stopwatch that measured time to the nearest 1/100th of a second, and the same investigator conducted all sprint trials during PRE and POST testing. The number of accurate shots was recorded.

#### Cognitive function

Immediately following the repeated sprints and shooting performance participants performed a modified version of the original Serial Sevens Test to analyze cognitive function (Hayman 1942). The test consisted of a 2-min timed written test in which participants were required to subtract the number 7 from a randomly generated four digit number, in order to measure how quickly and accurately they can compute a simple mathematical problem. The four digit number appeared on the top of the first column of a three column sheet of paper. Participants were provided the sheet of paper and asked to complete as many calculations as possible in the 2-min period. The answers to the calculations were written underneath the initial number. Regardless of answer provided, participants were then required to subtract the number 7 from that new number. Participants were not told if their answer was correct or not. The number of correct answers was recorded. Intraclass correlations for this assessment has been determined in our laboratory to be *R* > 0.81 (Wells et al. 2013). The test was conducted next to the firing range, and the range remained ‘hot’ (i.e., continuous shooting) throughout the 2-min test.

### Supplement schedule

The β-alanine supplement (CarnoSyn™) was obtained from Natural Alternatives International (San Marcos, CA, USA). Both the supplement and placebo were in tablet form and were similar in appearance. Participants in the supplement group were provided with 2 tablets of sustained-release β-alanine at a dose of (2 g per serving) three times per day (total β-alanine intake was 6 g per day) and subjects in the placebo group were provided with an equivalent amount of rice powder. Participants were instructed to consume the supplement following their meals with water. Each participant was provided with a bottle containing a week’s supply of tablets. All bottles were returned at the end of the week. All tablets left in the bottle were counted, recorded, and the next week’s bottle was provided to the participant. Supplementation occurred every day over a 30-day period.

### Statistical analysis

Data was analyzed using a 2 × 2 [treatment (BA, PL) × time (PRE, POST)] repeated measures analysis of variance. In the event of a significant F ratio, LSD post hoc comparisons were used. POST–PRE (∆) performance changes between groups were analyzed using an unpaired *t* test. Due to logistical issues only 10 of the 18 participants were able to have their muscle and brain carnosine concentrations assessed at the PRE assessment. Therefore, comparisons of ∆ carnosine content was analyzed using the non-parametric independent samples median test. An alpha level of *p* ≤ 0.05 was considered statistically significant for all comparisons. Spearman rank correlation analysis was used to examine the relationship between changes in carnosine content and performance measures. All data are reported as mean ± SD. Data were analyzed using SPSS v20 software (SPSS Inc., Chicago, IL).

## Results

Compliance for consuming the supplement or placebo was 100 %. No adverse events were reported from participants in either group during the duration of the study. Body mass of the participants did not change (*p* = 0.500) from PRE (74.2 ± 5.7 kg) to POST (74.1 ± 5.8 kg) assessments, and no differences were noted in comparisons between groups.

**Fig. 1:**
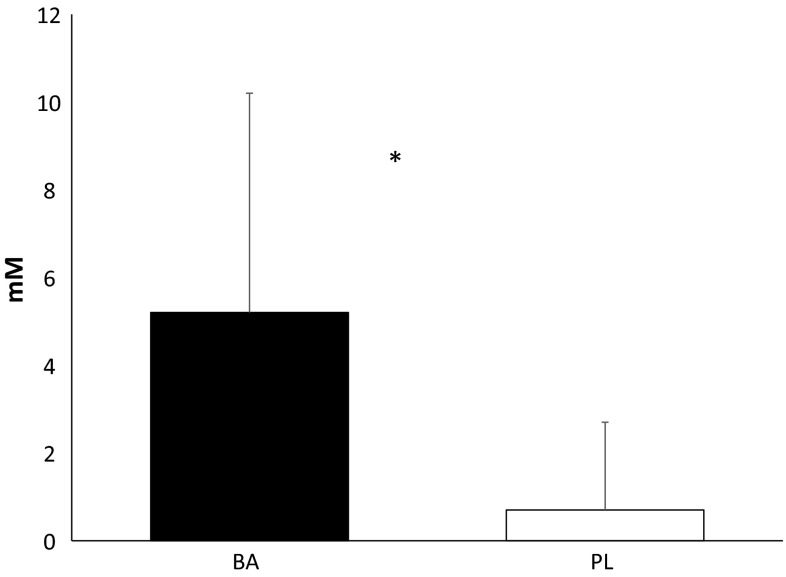
Changes in ∆ carnosine content in the gastrocnemius. All data are reported as mean ± SD. *Significant difference (*p* < 0.05) between groups, *BA* β-alanine, *PL* placebo

Comparisons in the ∆ carnosine content within the gastrocnemius muscle are shown in Fig. 1. Baseline carnosine content (6.7 ± 2.2 mM) in both groups was similar to that previously published (Baguet et al. 2010; Stellingwerff et al. 2012). Significant elevations (*p* = 0.048) from baseline was noted in BA compared to PL. Changes in the carnosine content of the gastrocnemius was moderately correlated to changes in fatigue rate in the 1-min sprint (*r* = 0.633, *p* = 0.060). Although these correlations were not statistically different they did indicate a trend towards a relationship between the change in muscle carnosine content and rate of fatigue. In regards to brain carnosine content, no changes were observable (*p* = 0.607) from PRE levels in either group. Considering the limit of detection in the 3 Tesla magnet is approximately 0.5 mM, it is likely that there was not a sufficient degree of sensitivity to discern any changes in brain carnosine content.

Comparisons between BA and PL in the measures examined during the 2.5 km run are described in Table 1. No significant differences were noted between the groups in the time for the 2.5 km run (*p* = 0.866), average velocity (*p* = 0.944) and average heart rate (*p* = 0.122). In addition, no significant differences were noted between the groups in the percent of distance run at low, (*p* = 0.873), medium (*p* = 0.502) and high intensity (*p* = 0.605).

**Table 1:** Performance variables during the 2.5 km run

Variable	Group	PRE	POST	*p* value
Time (s)	BA	624 ± 22.6	629 ± 23.8	0.866
	PL	633 ± 25.3	609 ± 36.4	
Average Velocity (ms^−1^)	BA	3.97 ± 0.17	4.09 ± 0.25	0.944
	PL	3.94 ± 0.14	4.05 ± 0.24	
Average heart rate (beats min^−1^)	BA	156.8 ± 15.5	165.7 ± 6.1	0.122
	PL	162.1 ± 11.7	159.3 ± 11.4	
Distance run at low intensity (%)	BA	12.3 ± 12.0	10.7 ± 14.0	0.873
	PL	15.2 ± 12.6	12.3 ± 14.9	
Distance run at moderate intensity (%)	BA	69.8 ± 12.1	65.1 ± 14.8	0.502
	PL	71.1 ± 10.9	63.7 ± 14.7	
Distance run at high intensity (%)	BA	18.4 ± 12.4	23.9 ± 20.1	0.645
	PL	13.3 ± 9.2	23.9 ± 20.2	

All data are reported as mean ± SD

*BA* β-alanine group, *PL* placebo group

Results of all sprint performance measures are depicted in Table 2. During the 1-min sprint no significant difference (*p* = 0.723) was observed in the total distance run from PRE (310.0 ± 16.7 m vs. 310.7 ± 23.7 m) to POST (302.4 ± 21.2 m vs. 306.6 ± 17.2 m) in either BA or PL, respectively, and no between group differences were noted as well. Similarly, no significant changes in either group were noted in peak or mean velocity, fatigue rate and the distance run at 90 % of peak velocity. In addition, no between group differences were noted in any of the measured variables.

**Table 2:** Performance variables during the sprint protocols and serial subtraction test

Assessment	Variable	Group	PRE	POST	*p* value
1-min sprint	Peak velocity (ms^−1^)	BA	6.68 ± 0.36	6.57 ± 0.18	0.354
		PL	6.71 ± 0.48	6.77 ± 0.43	
	Average velocity (ms^−1^)	BA	5.21 ± 0.28	5.09 ± 0.36	0.535
		PL	5.19 ± 0.33	5.15 ± 0.29	
	Fatigue rate (%)	BA	32.5 ± 5.5	30.9 ± 6.7	0.893
		PL	32.2 ± 7.1	30.1 ± 5.4	
	Distance run at 90 % peak velocity	BA	69.5 ± 4.1	58.5 ± 7.7	0.199
		PL	66.0 ± 6.0	60.8 ± 7.5	
Repeat 30-m sprints	Average sprint time (s)	BA	7.42 ± 0.24	8.00 ± 0.29	0.780
		PL	7.43 ± 0.26	8.00 ± 0.22	
	Fatigue rate (%)	BA	91.6 ± 4.8	91.8 ± 3.0	0.432
		PL	93.8 ± 1.8	92.4 ± 3.2	
50-m casualty carry	Time (s)	BA	15.60 ± 1.16	15.34 ± 0.97	0.044
		PL	14.64 ± 0.73	14.86 ± 0.85	
Serial subtraction	Number of correct responses (#)	BA	27.1 ± 8.2	31.9 ± 7.9	0.022
		PL	26.0 ± 6.1	26.4 ± 8.5	

All data are reported as mean ± SD

*BA* β-alanine group, *PL* placebo group

**Fig. 2:**
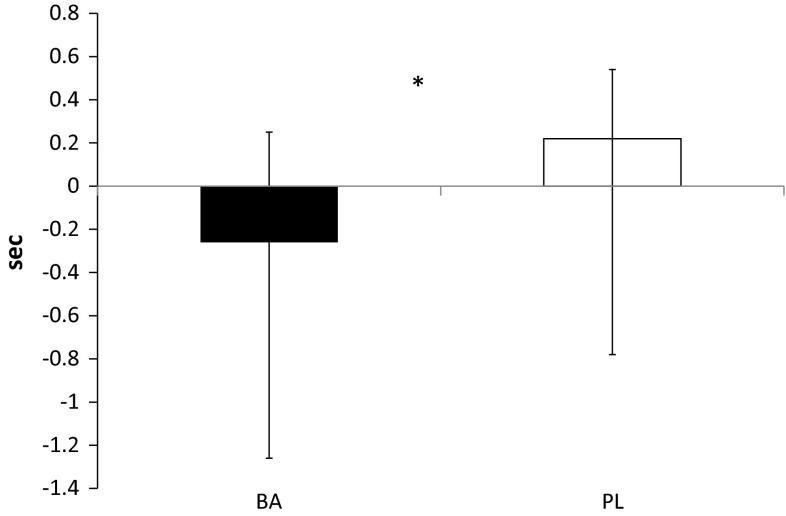
Changes in ∆ 50-m Casualty Carry. All data are reported as mean ± SD. *Significant difference (*p* < 0.05) between groups, *BA* β-alanine, *PL* placebo

**Fig. 3:**
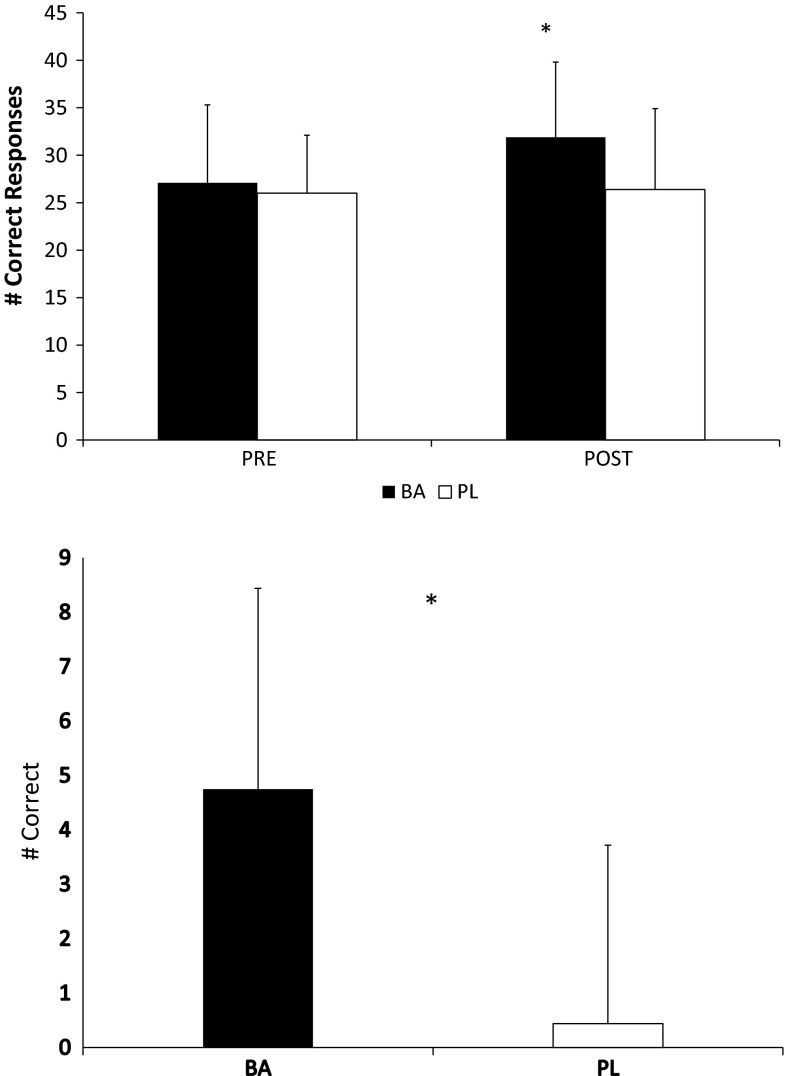
Changes in ∆ serial subtraction test. All data are reported as mean ± SD. *Significant difference (*p* < 0.05) between groups, *BA* β-alanine, *PL* placebo

Changes in the ∆ time for the 50-m casualty carry are depicted in Fig. 2. Participants in BA significantly (*p* = 0.044) improved their time for the sprint compared to PL. No differences were noted between the groups in average time or fatigue rate during the 30-m repeated sprint protocol (see Table 2). In addition, no differences from PRE to POST were noted in marksmanship for BA (14.0 ± 0.8 shots on target and 13.1 ± 2.3 shots on target, respectively) or PL (13.2 ± 1.7 shots on target and 12.9 ± 1.6 shots on target, respectively), and no between group differences (*p* = 0.755) were noted as well. However, a significant difference (*p* = 0.022) was observed in the serial subtraction test under stress (see Table 2; Fig. 3). Ingestion of β-alanine for 30-days appeared to significantly improve 50-m casualty carry time and serial subtraction test performance compared to placebo.

## Discussion

Results of this study demonstrate that 30-days of β-alanine ingestion was effective in elevating muscle carnosine content in the gastrocnemius muscle of elite combat soldiers during a period of high intensity training. In addition, the increase in β-alanine ingestion appeared to improve 50-m casualty carry time and cognitive performance (i.e., serial subtraction test). These results appear to confirm a recent investigation that supported the benefit of β-alanine ingestion on military personnel (Hoffman et al. 2014), and are consistent with current knowledge regarding elevations in muscle carnosine content.

Changes in muscle carnosine content in the gastrocnemius muscle was similar to that reported by Derave et al. (2007), but greater than that reported by Baguet et al. (2010). This difference may be related to training experience. Recent research has reported that trained muscle is more sensitive for synthesizing carnosine than untrained muscle (Bex et al. 2014). The study by Baguet et al. (2010) examined rowers who primarily exercised their upper body musculature, but the investigators measured carnosine content in the gastrocnemius muscle. In contrast, Derave et al. (2007) examined the gastrocnemius muscle group in athletes whose training and performance were focused primarily with the lower body. Similarly, the conditioning activity of the participants in this present study was primarily comprised of lower body activities (e.g., runs, marches and sprints).

Significant performance improvements were only noted in the 50-m casualty carry, but no significant changes were observed between BA and PL in the 2.5 km run, 1-min sprint or the repeated sprints and shooting. Based upon the physiological role of carnosine in muscle, these results are not surprising. Although elevations in muscle carnosine may enhance the ability to perform high intensity interval training that can indirectly enhance endurance performance (Smith et al. 2009), the limited number of studies examining β-alanine supplementation and traditional endurance training have not shown any efficacy in β-alanine ingestion and endurance performance (Hoffman et al. 2014; Jordan et al. 2010). The inconsistency seen in the high intensity performance measures supports the results of a recent meta-analysis that indicated that greatest ergogenic potential for elevated muscle carnosine concentrations occurs during high intensity exercise lasting 60–240 s in duration (Hobson et al. 2012).

The lack of any effect of BA ingestion on repeated sprint performance is similar to previous studies that used similar supplement dosages and duration of use in repeated sprint activity (Saunders et al. 2012; Sweeney et al. 2010). The duration of each sprint (7.43 ± 0.24 s) and the relatively low total number of sprints (e.g., five) was unlikely to generate a significant decline in muscle pH. The longer duration 50-m casualty carry though did result in a significant performance improvements for BA compared to PL. Following the 30-day supplement period, participants consuming β-alanine performed the sprint faster than those participants consuming the placebo. Although the duration of the sprint ranged from 13.72 to 17.18 s the added resistance provided by sprinting with a manikin in dirt and sand added a significant stress to the anaerobic energy system. The 60 kg manikin was approximately 81 % of the body mass of the average participant. A load of this magnitude has been shown to significantly enhance the metabolic cost associated with activity (Knapik et al. 2004), and is a stress commonly reported among infantry soldiers who carry between 29 and 60 kg in their backpacks during various military specific tasks (Nindl et al. 2013).

No significant differences were noted between BA and PL in any performance variable measured during the 1-min sprint. However, the moderate correlation (*r* = 0.633) noted between the change in muscle carnosine content and fatigue rate during sprint is consistent with previous studies examining fatigue rate and β-alanine supplementation (Derave et al. 2007; Stout et al. 2006). Regardless, no differences were noted between the groups in peak velocity, mean velocity, distance run for one-min, distance run at 90 % of peak velocity or fatigue rate during the 1-min sprint. Although the duration of the sprint was 1 min, this is at the low end of what is suggested to be the threshold for performance efficacy regarding β-supplementation (Hobson et al. 2012). It is likely that this range of performance times is specific to the training program utilized. Recommendations regarding β-alanine use is generally focused on the anaerobic athlete (Hoffman et al. 2012; Sale et al. 2010), however the physical preparation of the tactical athlete examined in this present study was focused primarily on endurance activity. Although many of the job task requirements of the combat soldier have been determined to be anaerobic in nature (Knapik et al. 2009), the training program of many combat soldiers are not job task specific (Nindl et al. 2013). It is likely that the non-anaerobic and/or non-competitive athlete may be limited in their ability to perform prolonged sprinting. Further analysis of the GPS data during the 1-min sprint revealed that the percentage of time spent sprinting (>6.11 ms^−1^) was similar between the groups, but only 15.3 ± 7.3 % of the total time recorded was actually considered to be at a rate consistent with a sprint.

In contrast to a recent study demonstrating improved marksmanship and target engagement speed following 4-weeks of β-alanine ingestion (Hoffman et al. 2014), this present study was unable to observe any between group differences. However, the shooting protocol between the studies was quite different. In the previous study, participants were required to overcome a misfire in their weapon and then continue to direct fire at their target 40-m away as rapidly as possible. A 24.8 % difference was noted in the time required to deliver 10 shots onto the target. In this study, participants fired three times on target between each sprint. There were no pre-arranged misfires and each participant had 5-s to deliver a volley of three shots to a target of shorter distance (30-m). This was performed to simulate close quarters combat in which soldiers move rapidly from cover to cover exchanging fire. Previous research has suggested that even in a fatigued state shooting accuracy can be maintained (Nibbeling et al. 2013), but shooting distance may change. In this present study, distance of the shooting remained consistent throughout the protocol. In addition, the previous study demonstrating improved marksmanship skills with β-alanine may have been related to improved cognitive ability under stress rather than reduced fatigue alone. Overcoming the misfire required the participant to focus and operate in a manner that determined the reason for the misfire, correct it and then continue shooting. This study did not include the same cognitive stress to the shooting protocol.

The current study reported significant improvements in cognitive performance, as assessed by the 2-min serial subtraction test. This contrasted with the lack of difference in this measure reported previously following 4-weeks of β-alanine ingestion (Hoffman et al. 2014). The differences between this present study and the previous investigation by Hoffman et al. (2014) may be in the manner in which the serial subtraction test was administered. In the previous study, participants sat down in a quiet area whereas in the present study, participants performed the test at the shooting range with continuous fire still being directed at targets. The participants in this present study were required to maintain their focus despite the active firing line that was occurring near them. The loud noise of the firing range coupled with the stress of performing mathematical problems may have contributed to a high level of anxiety within the participants. A recent study has indicated that anxiety can significantly decrease cognitive performance, and specifically mathematical skills in infantry soldiers (Nibbeling et al. 2014). The results of this present study indicate that 30-days of β-alanine ingestion enhance cognitive function to a greater extent than a placebo.

The mechanism that was potentially hypothesized to enhance cognitive function as a result of β-alanine ingestion focused on elevating brain carnosine levels. Previous research has suggested that β-alanine intake can increase carnosine content in the brain, which appears to result in an anxiolytic effect (Murakami and Furuse 2010). However, this was shown in a murine model and has not been previously demonstrated in humans. To date, carnosine content in the human brain has been reported to exist in the olfactory bulb only (Boldyrev et al. 2013). This anatomical region of the brain is primarily responsible for sense of smell, and the potential ergogenic effects related to carnosine levels in the olfactory bulb are not well understood. However, the parieto-occipital region though, which was examined in the present study, is responsible for processing visual and sensory information that is critical in the decision-making processes for the tactical athlete. However, to our knowledge no studies have been published examining the ability of β-alanine ingestion to increase carnosine levels in this region of the brain. This is likely related to the limitations of existing technology which makes it difficult to determine whether the peak on the MRS spectrum of the brain is carnosine only or also homocarnosine. Homocarnosine is a histidine containing dipeptide consisting of histidine and GABA and is thought to be more prevalent in the human brain than carnosine (Boldyrev et al. 2013). The impetus to investigate brain carnosine content in this study was predicated on the assumption that any changes in the spectrum would be attributed solely to an increase in carnosine as a result of β-alanine ingestion. Considering that the participants garrisoned together and were provided the same meals during the entire study, the assumption was that any change in the peak area at ~8.0 ppm of the spectrum in the brain measures could be only related to supplement ingestion. Considering that no change was noted between the groups it is likely that the sensitivity of MRS was not sufficient to detect carnosine changes that are of smaller magnitude than that seen in skeletal tissue.

A potential mechanism that can explain the improved cognitive function observed in this study can thus be only left to speculation. One possibility is that increases in carnosine may have elevated BDNF. BDNF is the most prevalent neurotrophin in the central nervous system where it supports existing neurons and promotes neuronal growth (Park and Poo 2012). Murakami and Furuse (2010) have demonstrated that increases in brain carnosine can elevate BDNF concentrations resulting in greater anxiolytic activity. Another possible role of carnosine in the brain has suggested that it acts as a reservoir for histidine, which is a precursor for histamine (Zhu et al. 2007). Decreases in histidine has been demonstrated to reduce brain histamine levels and subsequently increase anxiety-like behaviors (Yoshikawa et al. 2014), reduce cognitive function (Dai et al. 2007) and contribute to stress and depression (Yanai et al. 1998). When histamine levels are elevated levels of anxiety appear to be reduced (Endou et al. 2001), and memory task capability appears to be maintained (Benetti and Izquierdo 2013). It is possible that when carnosine is synthesized in the brain, it is quickly hydrolyzed to histidine and β-alanine by the enzyme carnosinase. Two isoforms of the enzyme were discovered with one form specifically found in the liver and brain (Jackson et al. 1991). However, others have suggested that an increased expression of carnosinase in the brain may actually have greater pathophysiological implications by decreasing carnosine levels and increasing risk of oxidative stress (Bellia et al. 2009). Clearly, additional work is needed in our understanding of carnosine synthesis in the brain from β-alanine ingestion.

In conclusion, the results of this study indicate that 30 days of β-alanine ingestion in soldiers of an elite combat unit can increase muscle carnosine content and improve 50-m casualty carry time. Further, changes in muscle carnosine content were moderately correlated to changes in fatigue rate during prolonged sprint activity. Although cognitive performance under stressful conditions were significantly greater in participants consuming β-alanine compared to placebo, the current study methods were unable to detect any change in brain carnosine levels, thus the precise mechanism underlying these effects remains elusive.
